# Consequences of chronic kidney disease in chronic obstructive pulmonary disease

**DOI:** 10.1186/s12931-019-1107-x

**Published:** 2019-07-12

**Authors:** Franziska C. Trudzinski, Mohamad Alqudrah, Albert Omlor, Stephen Zewinger, Danilo Fliser, Timotheus Speer, Frederik Seiler, Frank Biertz, Armin Koch, Claus Vogelmeier, Tobias Welte, Henrik Watz, Benjamin Waschki, Sebastian Fähndrich, Rudolf Jörres, Robert Bals, on behalf of the German COSYCONET consortium

**Affiliations:** 1 Department of Internal Medicine V - Pulmonology, Allergology Critical Care Care Medicine, Saarland University Hospital, Homburg, Germany; 2 Department of Internal Medicine IV – Nephrology, Saarland University Hospital, Homburg, Germany; 3 Institute for Biostatistics, Hannover Medical School, Hannover, Germany; 4 Department of Medicine, Pulmonary and Critical Care Medicine, University Medical Center Giessen and Marburg, Philipps-University Marburg, Member of the German Center for Lung Research (DZL), Marburg, Germany; 5 Clinic for Pneumology Hannover Medical School, Member of the German Center for Lung Research, Hannover, Germany; 6 Pulmonary Research Institute at LungenClinic Grosshansdorf Airway Research Center North, Member of the German Center for Lung Research, Grosshansdorf, Germany; 7 Institute and Outpatient Clinic for Occupational, Social and Environmental Medicine, Munich, Germany

**Keywords:** Chronic obstructive pulmonary disease, Chronic kidney disease, Patient-centered outcomes, Cohort study

## Abstract

**Background:**

The combination of chronic obstructive pulmonary disease (COPD) and chronic kidney disease (CKD) is associated with a higher prevalence of comorbidities and increased mortality. The impact of kidney function on patient-centered outcomes in COPD has not been evaluated.

**Methods:**

Patients from the German COPD and Systemic Consequences - Comorbidities Network (COSYCONET) cohort COPD were analysed. CKD was diagnosed if the estimated glomerular filtration rate (eGFR) measurements were < 60 mL/min/1.73m^2^ at study inclusion and six month later. The effect of CKD, on comorbidities, symptoms [modified British Medical Research Council dyspnoea scale], physical capacity [six-minute walk test, and timed up and go] and St George’s Respiratory Questionnaire were analysed. Restricted cubic spline models were used to evaluate a nonlinear relationship between eGFR with patient-centered outcomes, cox survival analysis was applied to evaluate mortality.

**Results:**

2274 patients were analysed, with CKD diagnosed in 161 (7.1%). Spline models adjusted for age, gender, BMI, FEV_1_ and cardiovascular comorbidities revealed independent associations between eGFR with modified British Medical Research Council dyspnoea scale, St George’s Respiratory Questionnaire, (*p* < 0.001 and *p* = 0.011), six-minute walk test (*p* = 0.015) and timed up and go (p < 0.001). CKD was associated with increased mortality, independently from for other cardiovascular comorbidities (hazard ratio 2.3; p < 0.001).

**Conclusion:**

These data show that CKD is a relevant comorbidity in COPD patients which impacts on patient-centered outcomes and mortality.

**Trial registration:**

NCT01245933

**Electronic supplementary material:**

The online version of this article (10.1186/s12931-019-1107-x) contains supplementary material, which is available to authorized users.

## Introduction

Chronic obstructive pulmonary disease (COPD) and chronic kidney disease (CKD) affect a large number of patients. The World Health Organization estimates COPD to become the 3rd leading cause of mortality worldwide in 2030 [1]. CKD, defined by abnormalities of kidney structure or function for more than 3 months [2], affected 14.8% of the U.S. adult general population in 2011–2014 [3]. Cigarette smoking and increasing age are risk factors for the development of both COPD and CKD [4–6], with systemic inflammation as an extrapulmonary manifestation of COPD potentially increasing the risk of comorbid CKD [7]. This combination of COPD and CKD is independently associated with a higher prevalence of other comorbidities (especially cardiovascular) and increased mortality [8, 9].

The presence of a number of comorbidities has been shown to correlate with limitations of exercise capacity in COPD patients. Cardiovascular dysfunction is a well-known predictor of a limited functional capacity and health status [10]. Whether CKD and kidney function have a role for functional limitations independent of established cardiovascular disease is currently unknown. The German COPD and Systemic Consequences - Comorbidities Network (COSYCONET) is a multicentre prospective cohort study investigating the interaction of COPD, comorbidities and systemic inflammation [11]. The present study aimed to analyse the relationship between COPD, CKD and estimated glomerular filtration rate (eGFR), focusing on patient-centered outcomes and mortality.

## Methods

### Study population

COSYCONET recruited patients age ≥ 40 years and with a diagnosis of COPD or symptoms of chronic bronchitis who were available to attend repeated study visits up to 18 months. The characteristics of the cohort have been described previously [11]. A total of 2741 participants were recruited from September 2010 to December 2013 in 31 study centres throughout Germany. The present study analysed data from the baseline visit and the first follow-up at 6 months. Mortality was assessed until November 2017.

### Definition and staging of chronic kidney disease

CKD was diagnosed by the estimated glomerular filtration rate, based on the Chronic Kidney Disease Epidemiology Collaboration (CKD-EPI) creatinine equation [12]. Patients with an eGFR < 60 mL/min/1.73 m^2^ at study inclusion and at the six month visit were considered as having CKD, as per the Kidney Disease Outcome Quality Initiative (KDOQI) guidelines [2]. CKD category 1 and 2 (eGFR ≥90 and 60–89 mL/min/1.73 m^2^, respectively), reflecting normal or mildly decreased kidney function, were combined into the category ‘no CKD’. CKD categories were defined as follows: CKD category 3a (eGFR 45–59 mL/min/1.73 m^2^), category 3b (eGFR 30–44 mL/min/1.73 m^2^), category 4 (eGFR 15–29 mL/min/1.73 m^2^) and category 5 (eGFR < 15 mL/min/1.73 m^2^). Patients with missing laboratory values at one or both time points were excluded from the first part of the present analyses.

### Pulmonary function, GOLD classification

All pulmonary function tests (i.e. forced spirometry, body plethysmography and diffusion capacity) were performed 45 min after inhalation of 400 μg salbutamol and 80 μg ipratropium bromide according to current recommendations [13–17].

Due to the above mentioned inclusion criteria there were also some patients with a FEV_1_/FVC ratio above 70% at baseline. These patients were described as GOLD Stage 0. This group was defined as having a FEV_1_/FVC ratio > 70% and (i) having a doctor’s diagnosis of chronic bronchitis and/or (ii) indicating a severity of cough of at least 3 in the respective COPD Assessment Test (CAT) item and/or (iii) indicating a severity of phlegm of at least 3 in the respective CAT item [11].

### Comorbidities

All participants underwent structured interviews to identify other comorbidities. The overall comorbid burden was summarised in a main comorbidity index (MCI). The MCI depicts a non-weighted summary score of the 34 following conditions: allergic diseases, arrhythmia, asthma, cancer, cirrhosis of the liver, coronary artery disease, chronic bronchitis, epilepsy, gastritis, gastroesophageal reflux, gout, heart failure, hepatitis, hypertension, hypothyroidism, hyperthyroidism or hyperparathyroidism, mental disorders, insulin-dependent diabetes mellitus, multiple sclerosis, myocardial infarction, non-insulin-dependent diabetes mellitus, osteoarthrosis, osteoarthritis, osteoporosis, peptic ulcer, parkinson disease, peripheral artery disease, peripheral neuropathy, pulmonary fibrosis, renal colic or renal calculi, sarcoidosis, sleep apnea, stroke and venous thrombosis. The MCI was calculated by counting each item with 1 point. A summarised assessment of cardiovascular comorbidity was performed in a similar manner using the cardiovascular index (CVI), which includes the five cardiovascular items hypertension, coronary artery disease, myocardial infarction, arrhythmia and stroke. Patients with a CVI of ≥1 point were considered as having cardiovascular comorbidities.

### Measurements of symptoms, functional status, exercise capacity and health status

Severity of dyspnoea was assessed using the modified British Medical Research Council dyspnoea scale (mMRC) [18]. The COPD related symptom load was assessed by the COPD Assessment Test (CAT) [19]. Functional status and exercise capacity were assessed by the ‘timed up and go’ (TuG) and the six-minute walk test (6MWT). The ‘timed up and go’ measures the time taken for the patient to rise from a chair, walk 3 m, turn, walk back, and sit down again [20]. The six-minute walk test was performed as described in the former American Thoracic Society (ATS) guidelines [21]. COPD specific health status was measured by the St George’s Respiratory Questionnaire (SGRQ) [22]. Quality of life was measured by the EuroQoL 5-dimension (EQ-5D) Questionnaire.

### Statistical analysis

The association of CKD with functional, laboratory values and other comorbidities were analyzed using group comparisons. We described categorical data using frequencies and percentages. For continuous data we used means (standard deviations), those values which were markedly different from normal distribution are presented as median (interquartile range). Comparisons between the “CKD and the ‘no CKD’ group were performed by Fisher’s exact test or X^2^ test, as appropriate in case of categorical variables, t-tests or Wilcoxon test were used for continuous variables as appropriate. Multivariate regression models with included established risk factors (e.g. age, sex, BMI, FEV1% pred.) were used for analysis of the impact of CKD for different numeric variables. Analysis was performed in SAS 9.3 and results were considered statistically significant for *P* values less than 0.05. Because of the non-linear association between mMRC, TuG, 6MWT, SGRQ, FEV1, BMI and eGFR, we analyzed non-linear associations between the aforementioned parameters and eGFR from the first visit by using restricted cubic splines of eGFR with three knots. Knots were placed at 59.6 ml/min, 84.8 ml/min, and 100.8 ml/min which corresponds to the 10th, 50th, and 90th percentile of the eGFR values. Analyses were adjusted for age, sex, BMI, FEV1 (% pred.) and CVI score, where appropriate. Analyses were performed using STATA IC 15. Multivariate adjusted restricted cubic spline analyses were performed using the STATA package ‘postrcspline’. Cox analysis was used to characterize the impact of CKD on mortality with additional independent variables: BMI, sex, CVI, and FEV1% pred. Analysis was performed using SPSS version 24 (IBM, Armonk NY, USA).

## Results

### Study subjects and prevalence of CKD

After screening of all 2741 patients from the COSYCONET study cohort, 2274 were eligible for analysis of CKD. 467 patients with missing laboratory values at one or both of the two defined time points were excluded from the CKD part of the analysis. CKD was diagnosed in 161 of 2274 patients (7.1%). The majority of all patients (60.6%) were male, and the mean ± SD age was 65.0 ± 8.4 years. Among the 161 patients with CKD, 114 (70.8%) were category 3A, 43 (26.7%) were category 3B, and 4 (2.5%) were category 4. There were no patients with an eGRF < 15 mL/min/1.73 m^2^ or on Dialysis. The distribution of chronic kidney disease categories in the study population is presented in Table 1.

**Table 1:** Distribution of chronic kidney disease categories in the study population

Kidney function	CKD categories	eGFR (mL)	No. of Patients (%)
normal to mild reduced	1–2	> 60	2113 (92.9)
moderate reduced	3A	45–59	114 (5.0)
	3B	20–44	43 (1.9)
severely reduced	4	15–29	4 (0.2)
kidney failure	5	< 15 or on Dialysis	0 (0)

CKD categories were defined in accordance with the National Kidney Foundation–Kidney Disease Outcomes Quality Initiative (KDOQI) guideline Abbreviations: CKD Chronic Kidney Disease; eGFR estimated Glomerular Filtration Rate

### Patients characteristics

Patients with CKD were significantly older and had a significantly higher BMI than those with normal or mildly reduced kidney function (i.e. the ‘no CKD’ group) (Table 2). Compared with the ‘no CKD’ group, patients with CKD showed less residual volume, and were more likely to be classified to be in the lower GOLD stages (0.0073). There were no differences between the two groups in terms of other spirometric parameters, diffusion capacity, or oxygenation. The characteristics of the study population are presented in Table 2.

**Table 2:** Patient characteristics

	N	All	No CKD	CKD	*p* value
Age (years)	2274	65.0 ± 8.4	64.5 ± 8.3	72.2 ± 6.6	**< 0.0001**
Male	2274	1378 (60.6%)	1280 (60.6%)	98 (60.9%)	0.9471
BMI (kg/m^2^)	2272	27.2 ± 5.2	27.00 ± 5.2	28.7 ± 5.3	**< 0.0001**
Smoking history (PY) ^a^	2192	40.0 [16.5–63.8]	39.0 [16.5–63.0]	51.3 ± 43.3	0.0793
Lung function					
FVC (L)	2256	3.0 ± 1.0	3.0 ± 1.0	2.9 ± 0.9	0.1660
FVC (%pred)	2256	78.6 ± 18.9	78.7 ± 18.9	77.4 ± 18.4	0.3875
FEV_1_ (L) ^a^	2260	16 [1.1–2.1]	1.6 [1.2–2.1]	1.5 [1.1–2.0]	0.3162
FEV_1_ (%pred)	2260	57.0 ± 21.0	57.0 ± 20.9	56.6 ± 22.4	0.7416
ITGV (L)	2205	4.7 ± 1.3	4.7 ± 1.3	4.7 ± 1.3	0.4425
ITGV (%pred)	2205	143.5 ± 37.7	143.2 ± 37.5	147.1 ± 39.6	0.2098
RV (L)	2194	3.8 ± 1.23	3.8 ± 1.2	3.9 ± 1.3	0.1667
RV (%pred)	2134	167.42 ± 58.6	168.2 ± 58.8	156.1 ± 54.5	**0.0143**
TLC (L)	2189	7.1 ± 1.5	7.1 ± 1.5	7.1 ± 1.4	0.7341
TLC (%pred)	2189	115.5 ± 20.3	115.3 ± 20.2	117.9 ± 21.3	0.1474
TLCO (%)	2146	55.7 ± 21.8	55.9 ± 21.7	55.4 ± 23.1	0.7861
GOLD Classification					
Stage 0	2260	363 (16.1)	333 (15.8)	30 (18.6)	**0.0073**
Stage I	2260	182 (8.1)	166 (7.9)	16 (9.9)	
Stage II	2260	831 (36.7)	760 (36.2)	71 (44.10)	
Stage III	2260	706 (31.2)	666 (31.8)	40 (24.8)	
Stage IV	2260	178 (7.9)	174 (8.3)	4 (2.5)	
Blood gas analysis					
pH value	2213	7.4 ± 0.1	7.4 ± 0.1	7.4 ± 0.0	0.3847
PaO_2_ (mmHg)	2212	67.4 ± 9.2	67.3 ± 9.1	68.5 ± 10.8	0.1792
PacO_2_ (mmHg)	2212	37.7 ± 4.9	37.7 ± 4.9	37.6 ± 4.6	0.7490
HCO3 (mmol/L)	2211	24.3 ± 2.9	24.2 ± 2.9	24.4 ± 2.8	0.6371

Values are presented as mean ± standard deviations or number (%). Those values which were markedly different from normal distribution (^a^) are presented as median [interquartile range]. *p* ≤ 0.05 was considered statistically significant (bold)

*Abbreviations*: *BMI* body mass index, *PY* pack-years, *FEV1* forced expiratory volume in 1 s, *RV* residual volume, *TLC* total lung capacity, *ITGV* intrathoracic gas volume, *TLCO* transfer factor for carbon monoxide

### Comorbidity burden

Self-reported comorbidities were more frequent in the CKD group, in particular cardio- and cerebrovascular disease, peripheral artery disease (PAD), diabetes, gout and malignancies (Table 3). Furthermore, compared to the ‘no CKD’ group, patients with CKD were more likely to have higher CVI and MCI scores.

**Table 3:** Selected self-reported comorbidities

comorbidities	N	All	No CKD	CKD	*p* value
Hypertension	2206	1227 (55.7)	1112 (54.32)	115 (74.2)	**< 0.0001**
CAD	2200	343 (15.7)	306 (15.0)	39 (25.2)	**0.0008**
MI	2202	182 (8.3)	162 (7.9)	20 (12.9)	**0.0315**
Arrhythmia	1183	196 (16.7)	168 (15.2)	28 (36.8)	**< 0.0001**
Heart failure	1182	118 (10)	101 (9.1)	17 (22.4)	**0.0003**
Stroke	2202	92 (4.2)	80 (3.9)	12 (7.7)	**0.0242**
PAD	2202	255 (11.6)	226 (11.0)	29 (18.7)	**0.0046**
DM	2202	111 (5.0)	92 (4.5)	19 (12.3)	**< 0.0001**
Gout	2202	380 (17.2)	320 (15.6)	60 (38.7)	**< 0.0001**
Malignant tumour	2202	256 (11.6)	226 (11.0)	31 (20.0)	**0.0009**
Osteoporosis	2201	320 (14.5)	290 (14.1)	30 (19.4)	0.0787
Pathologic fracture	2202	100 (4.5)	93 (4.5)	7 (4.5)	0.9876
CVI (≥1)	2274	1398 (63.4)	1315 (62.2)	129 (80.1)	**< 0.0001**
MCI (≥5)	2274	1045 (47.4)	961 (45.9)	109 (67.4)	**< 0.0001**

*Abbreviations*: *CAD* coronary artery disease, *MI* myocardial infarction, *PAD* peripheral artery disease, *DM* Type I and Type II diabetes mellitus using insulin, *CVI* cardiovascular index, *MCI* main comorbidity index. Values are presented number (%). *p* ≤ 0.05 was considered statistically significant (bold)

### Laboratory testing

Haemoglobin was significant lower in patients with CKD compared with the ‘no CKD’ group. CKD patients presented significantly elevated blood glucose and glycosylated haemoglobin compared to the‘no CKD’ group. There were no differences between the two groups in term of leucocytes, C-reactive protein or cholesterol. Laboratory findings are summarized in the Additional file 1: Table S1.

### Measurements of symptoms, functional status, exercise capacity and health status

Patients with CKD had a significant higher mMRC values as compared to the ‘no CKD’ group. The COPD related symptom load as measured by the CAT showed no differences between the two groups (Table 4). Functional status and exercise capacity were reduced in CKD patients as they took significantly longer to complete the TuG as compared to the ‘no CKD’ group and the distance walked in 6 min was significantly shorter. COPD specific health status and quality of life showed no differences between the two groups (Table 4). Multivariate regression models with included established risk factors (e.g. age, sex, BMI, FEV1%pred) were used for analysis of the impact of CKD for different numeric variables (dyspnoea, functional status, exercise capacity and QOL). The effect of CKD on the distance walked in 6 min was independent from the effect of age, gender, BMI, FEV1 and CVI (point estimate, 17.6 m; 95% confidence interval, 0.8–34.4,*p* < .0001).

**Table 4:** Measurement of dyspnoea, COPD specific health status, quality of life, exercise capacity and physical activity

	N	All	No CKD	CKD	*p* value
mMRC	2260				**< 0.0001**
0		207 (9.2)	196 (9.3)	12 (7.5)	
1		1067 (47.2)	1014 (48.3)	53 (32.9)	
2		614 (27.2)	557 (26.5)	57 (35.4)	
3		353 (15.6)	317 (15.1)	36 (22.4)	
4		19 (0.9)	16 (0.8)	3 (1.9)	
CAT	2263	17.8 ± 7.2	17.8 ± 7.2	17.4 ± 7.0	0.5117
SGRQ	2259	41.7 ± 19.6	41.5 ± 19.5	44.0 ± 19.9	0.1196
EQ 5D	2266	0.8 ± 0.2	0.8 ± 0.2	0.8 ± 0.2	0.6427
6MWD	2225	424.7 ± 105.2	427.6 ± 104.3	385.9 ± 110.1	**< 0.0001**
TuG (sec.)	2224	6.9 ± 2.2	6.9 ± 2.2	7.5 ± 2.4	**0.0004**

*Abbreviations*: *mMRC* Modified British Medical Research Council dyspnoea scale, *CAT* COPD Assessment Test, *6-MWD* Six minute walk distance, *SGRQ* St George’s Respiratory Questionnaire, *EQ-5D* EuroQol- 5 dimension. Values are presented as N (%) or mean ± SD. *p* ≤ 0.05 was considered statistically significant (bold)

### Restricted cubic spline models

**Fig. 1:**
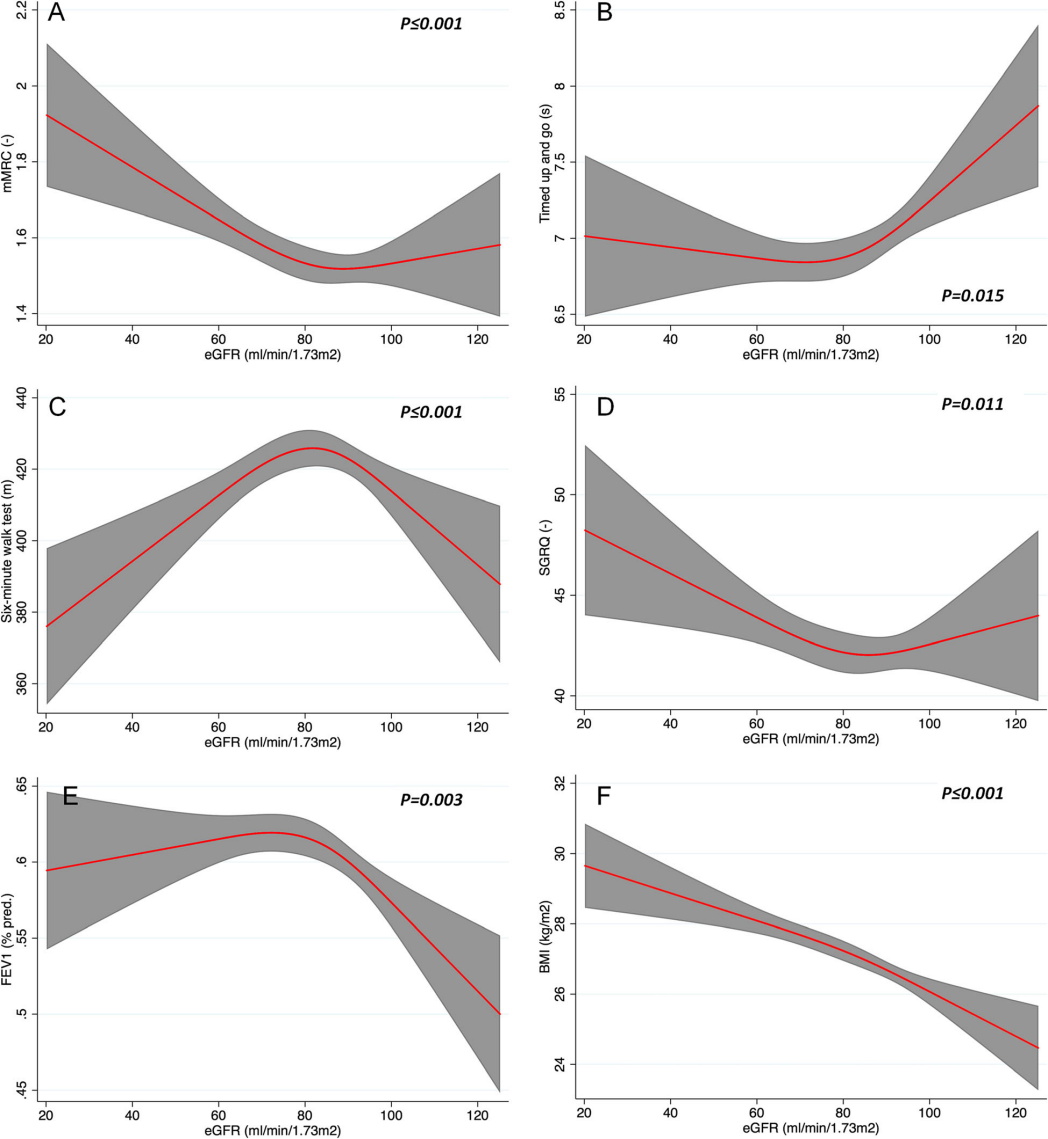
Restricted cubic spline plots of the association of eGFR with (**a**) Modified British Medical Research Council dyspnoea scale; mMRC, (**b**) timed up and go TuG in seconds, (**c**) six-minute walk test in meters, (**d**) St George’s Respiratory Questionnaire; SGRQ, (**e**) forced expiratory volume in 1 s; FEV1 in % predicted and (**f**) body mass index; BMI. The red line indicates the estimated change of mMRC, TuG, 6MWT, SGRQ, FEV1 (%pred.) with the respective 95% confidence interval (gray area). **a-d** are adjusted for age, gender, BMI, FEV1 (% pred.) and cardiovascular comorbidity (CVI 1–5). **e** and **f** for age, sex, cardiovascular comorbidity (CVI score) and either BMI or FEV1 (% pred.)

Spline models adjusted for age, sex, BMI, FEV1 (% pred.) and cardiovascular comorbidity (CVI score) were performed to analyse the non-linear association of eGFR with dyspnea, functional status (FS), exercise capacity (EC) and quality of life (QoL). These models revealed independent relationships of eGFR with mMRC, TuG, 6MWT, and SGRQ. Figure 1a and d show eGFR as an independent predictor of mMRC (*p* < 0.001) and SGRQ (*p* = 0.011) with j-shaped associations. Figure 1c shows an u-shaped relationship of eGFR with 6MWT (p < 0.001), while the association of eGFR with the timed up and go is reverse j-shaped (*p* = 0.015, Fig. 1b). Figure 1e and f show spline plots for the association of eGFR with FEV1 (% pred.) and BMI adjusted for age, sex, cardiovascular comorbidity (CVI score) and either BMI or FEV1 (% pred.). These models reveal an association of lower FEV1% pred. and BMI with higher eGFR values (*P* = 0.003 and 0.001 respectively)

### Impact of CKD mortality

**Fig. 2:**
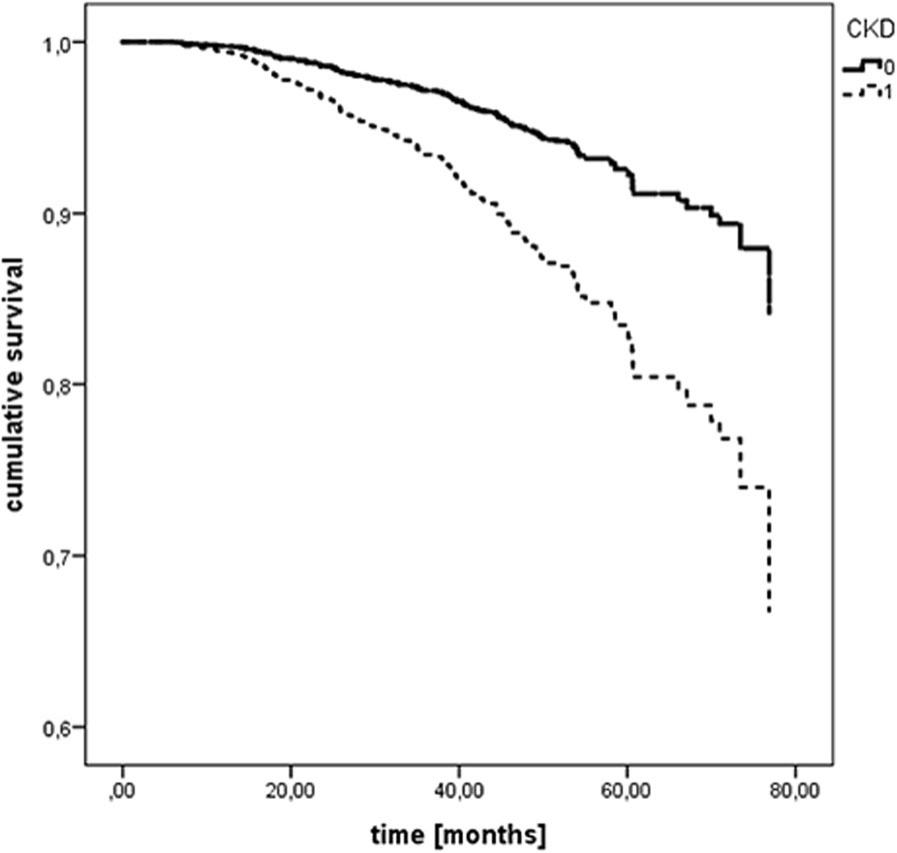
Cox analysis with BMI, sex, packyears, CVI, and FEV1% pred. as cofounders showed that CKD is significantly associated with mortality

To investigate whether COPD patients with comorbid CKD have an increased risk of dying, we performed Cox regression analysis with age, BMI, sex, packyears, CVI, and FEV1% pred. as cofounders and found that CKD is significantly associated with increased mortality (Fig. 2). This association was stable also from models that included the individual comorbidities or risk factors (data not shown). The hazard ratios (confidence intervals, *p* value) were: CKD, 2.35 (1.52–3.63, p = < 0.001); sex (male) 1.49 (1.03–2.14, *p* = 0.032), FEV1% pred. 0.96 (0.95–0.97, *p* = 0.000); age 1.09 (1.06–1.11, p = 0.000). No significance was found for CVI and BMI.

## Discussion

The present study characterized patients with comorbid COPD and CKD from the German COSYCONET study cohort. This is to our knowledge the first study analysing the effects of comorbid CKD on patient-centered outcomes in COPD. COPD Patients with CKD were more likely to have additional comorbidities, reported increased dyspnea, and had a significantly reduced exercise capacity compared with the ‘no CKD’ group. Spline models adjusted for age, gender, BMI, FEV1 (% pred.) and cardiovascular comorbidity revealed independent nonlinear associations of eGFR with dyspnoea, functional status, exercise capacity and health status. CKD was furthermore a predictor for mortality independently from other cardiovascular comorbidities.

There are several studies focusing on the prevalence of CKD in patients with COPD, conducted in a range of populations [23–29]. Most of these studies are single-center studys with a small sample size One recent meta-analysis by Gaddam and colleagues showed an increased prevalence of CKD in patients with COPD even after adjustment for co-variates including age, gender, BMI and smoking status, thus suggesting an independent association of CKD with COPD [30]. The overall CKD prevalence in our study population was 7.1%. This finding is consistent with that in other COPD populations reporting a CKD prevalence of 4–8% [23, 25, 29]. Systemic inflammation might be one linking element between these two conditions [7].

In the present study, mMRC scores were higher in patients with CKD and spline interpolations revealed an independent inverse association of eGFR with mMRC. Increased mMRC values in turn are linked with reduced physical activity levels in patients with COPD [31]. The higher level of dyspnoea in patients with comorbid CKD and COPD was also associated with reduced exercise capacity as measured by the six minute walk test. Spline interpolations for the association of eGFR with 6MWD distance showed a linear independent association if eGFR values were below 60 ml/min/1,73m^2^. This relationship was also shown for eGFR and COPD specific health status measured by the SGRQ if kidney function were reduced. However those patients with normal kidney function showed mixed outcomes for mMRC, timed up and go, six minute walk test and SGRQ. Especially those patients with high eGFR (> 90 ml/min/1.73m^2^) values presented more symptoms and inferior performance. The combination of high eGFR values and unfavourable outcomes in apparently healthy subjects was described as renal hyperfiltratration (RH). The pathogenesis of RH is still poorly understood, but there are associations with hypertension, diabetes, obesity and smoking [32]. Renal hyperfiltratration was shown as an independent predictor of chronic cardiopulmonary diseases and all-cause mortality [33]. This is commonly regarded as an overestimation of GFR because of muscle wasting in a high risk group. Our data support this theory as low FEV_1_ and BMI were independently associated with high eGFR values in our cohort. COPD related inactivity and sarcopenia might be on explanation for these findings, however the BMI values were still in the normal range and the BMI includes no information on body composition.

We also assessed whether CKD is associated with increased mortality. CKD is closely associated with cardiovascular diseases and an independent risk factor for death [34, 35]. This finding is in agreement with earlier studies that used health care system data [8, 36] and showed that COPD increased risk of death in CKD patients. CKD also increases the mortality risk in patients with acute exacerbations of COPD [37]. Our study suggests that these negative outcomes might be mediated by an impact of CKD on symptoms, functional status and exercise capacity. The effects of CKD on exercise capacity cannot simply be explained by the higher frequency of these comorbidities, but suggest that CKD per se has a negative effect on exercise capacity. The underlying mechanisms for this finding are likely complex and include increased systemic inflammation, (patho-)physiological interaction between lung and kidney, or network effects between several comorbidities including cardiovascular diseases. CKD contributes substantially to other common systemic manifestations of COPD such as malnutrition, muscle wasting, anaemia [38], osteoporosis and cardiovascular disease [38, 39], which in total negatively affect exercise capacity [21] and therefore, might explain the results of our study.

The present study has some limitations: The presence of comorbidities was based on patients’ reports. Other limitations are mainly related to the limited sample size in the CKD categories. The majority of patients within this category had only moderate kidney impairment, probably because these are the ones that are more willing to participate into cohorts, which might lead to a selection bias.

Our results from eGFR spline interpolations suggest that there is an increasing impact on dyspnoea, exercise capacity and health status with increasing kidney impairment. We therefore speculate that a higher proportion of CKD category 4 and 5 patients would have led to more pronounced differences between the two categories.

## Conclusion

CKD is a frequent finding in COPD patients and possibly an important contributor to the comorbidome of the disease as well as to many important disease outcomes, including mortality. Spline models showed a nonlinear association of eGFR on different patient-centered outcomes, CKD but also high eGFR values might be predictors for inactivity and progressive deconditioning in COPD. Interventions that increase physical activity levels might play a key role to improve outcomes in these special groups of patients. CKD is therefore a relevant COPD comorbidity, and there is an urgent need for more information to improve outcome in this high risk group of patients.

## Additional file

Additional file 1: **Table S1.** Laboratory values. (DOCX 18 kb)

## Data Availability

The data are part of the German COPD cohort COSYCONET (http://www.asconet.net) and available upon request.

## Abbreviations

6MWT: Six-minute walk test
BMI: Body mass index
CAD: Coronary artery disease
CAT: COPD assessment test
CKD: Chronic kidney disease
CKD-EPI: Chronic kidney disease epidemiology collaboration
COPD: Chronic obstructive pulmonary disease
CRP: C-reactive protein
CVI: Cardiovascular index
DM: Diabetes mellitus
EC: Exercise capacity
eGFR: Estimated glomerular filtration rate
EQ-5D: EuroQol- 5 dimension
FEV_1_: Forced expiratory volume in 1 s
FS: Functional status
HbA1c: Glycosylated haemoglobin
HDL: High density lipoprotein
ITGV: Intrathoracic gas volume
KDOQI: National kidney foundation–kidney disease outcomes quality Initiative
LDL: Low density lipoprotein
MCI: Main comorbidity index
MI: Myocardial infarction
mMRC: Modified british medical research council dyspnoea scale
PAD: Peripheral artery disease
PY: Pack-years
QoL: Quality of life
RV: Residual volume
SGRQ: St George’s Respiratory Questionnaire
TLC: Total lung capacity
TLCO: Transfer factor for carbon monoxide.
TuG: Timed up and go
