# Supplementary material for: Consequences of chronic kidney disease in chronic obstructive pulmonary disease
Source: Respir Res. 2019 Jul 12;20:151. doi: 10.1186/s12931-019-1107-x (PMC6626422; doi:10.1186/s12931-019-1107-x)
Supplement: Supplementary file 1 — Table S1. Laboratory values. (DOCX 18 kb) [file 12931_2019_1107_MOESM1_ESM.docx]

**Additional file 1**

**Consequences of chronic kidney disease in chronic obstructive pulmonary disease**

Franziska C. Trudzinski, Mohamad Alqudrah, Albert Omlor, Stephen Zewinger, Danilo Fliser, Timotheus Speer, Frederik Seiler, Frank Biertz, Armin Koch, Claus Vogelmeier, Tobias Welte, Henrik Watz, Benjamin Waschki, Sebastian Fähndrich, Rudolf Jörres and 1Robert Bals on behalf of the German COSYCONET consortium

**Table S1: Laboratory values**

| **Laboratory values** | **N** | **All** | **No CKD** | **CKD** | **P** |
| --- | --- | --- | --- | --- | --- |
| **Leucocytes (10^3/µL)** | 2262 | 7.9 ± 2.4 | 7.8 ± 2.3 | 8.3 ± 2.5 | **0.0093** |
| **Haemoglobin( mmol/L)** | 2194 | 9.1 ± 0.9 | 9.1 ± 0.8 | 8.7 ± 1.0 | **<0.0001** |
| **Haematocrit (%)** | 2194 | 43.5 ± 3.7 | 43.6 ± 3.6 | 42.0 ± 4.5 | **<0.0001** |
| **Creatinine (mg/dL)** | 2206 | 0.9 ± 0.2 | 0.9 ± 0.2 | 1.3 ± 0.3 | **<0.0001** |
| **Urea (mmol/L)** | 2201 | 5.2 ± 2.1 | 5.0 ± 1.8 | 8.1 ± 3.6 | **<0.0001** |
| **Glucose (mg/dL)** | 2152 | 101.8 ± 29.7 | 101.8 ± 28.7 | 110.25 ± 40.01 | **0.0069** |
| **HbA1c (mmol/mmol)** | 2170 | 41.1 ± 7.6 | 40.9 ± 7.5 | 43.9 ± 8.6 | **<0.0001** |
| **CRP (mg/dL)** | 2201 | 0.1 ± 2.3 | 1.0 ± 2.3 | 1.2 ± 2.6 | 0.254 |
| **Cholesterol (mg/dL)** | 2200 | 215.4 ± 43.2 | 215.7 ± 42.9 | 211.7 ± 46.3 | 0.2623 |
| **HDL (mg/dL)** | 2176 | 64.0 ± 21.3 | 64.2 ± 21.0 | 60.8 ± 23.8 | 0.0886 |
| **LDL (mg/dL)** | 2163 | 127.8 ± 37.9 | 128.0 ± 37.8 | 124.7 ± 39.1 | 0.2933 |

Abbreviations: HbA1c glycosylated haemoglobin; CRP C-reactive protein; LDL low density lipoprotein; HDL high density lipoprotein. Values are presented as mean ± STD.
